# Supplementary figures and images for: Simulation-based inference for efficient identification of generative models in computational connectomics
Source: PLoS Comput Biol. 2023 Sep 22;19(9):e1011406. doi: 10.1371/journal.pcbi.1011406 (PMC10550169; doi:10.1371/journal.pcbi.1011406)

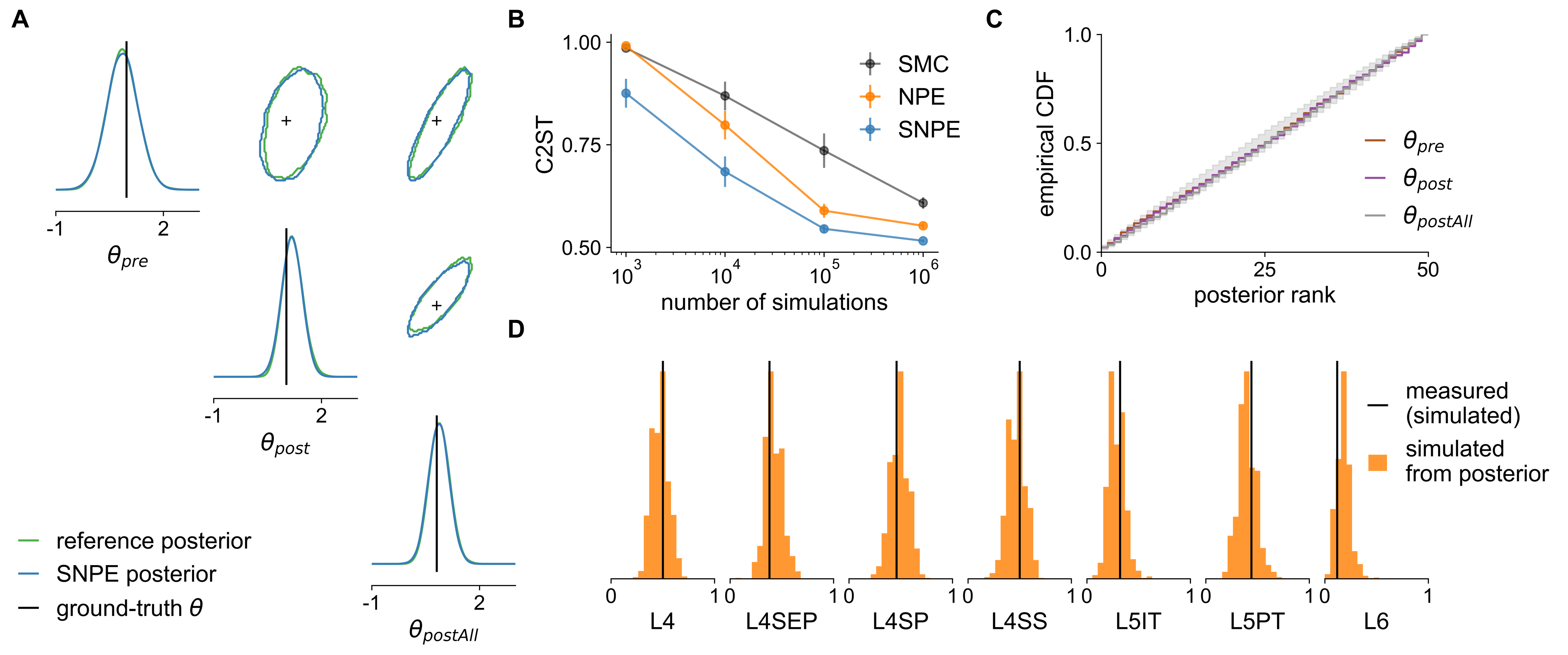

Supplement: S1 Fig — (TIF) [file pcbi.1011406.s001.tif]

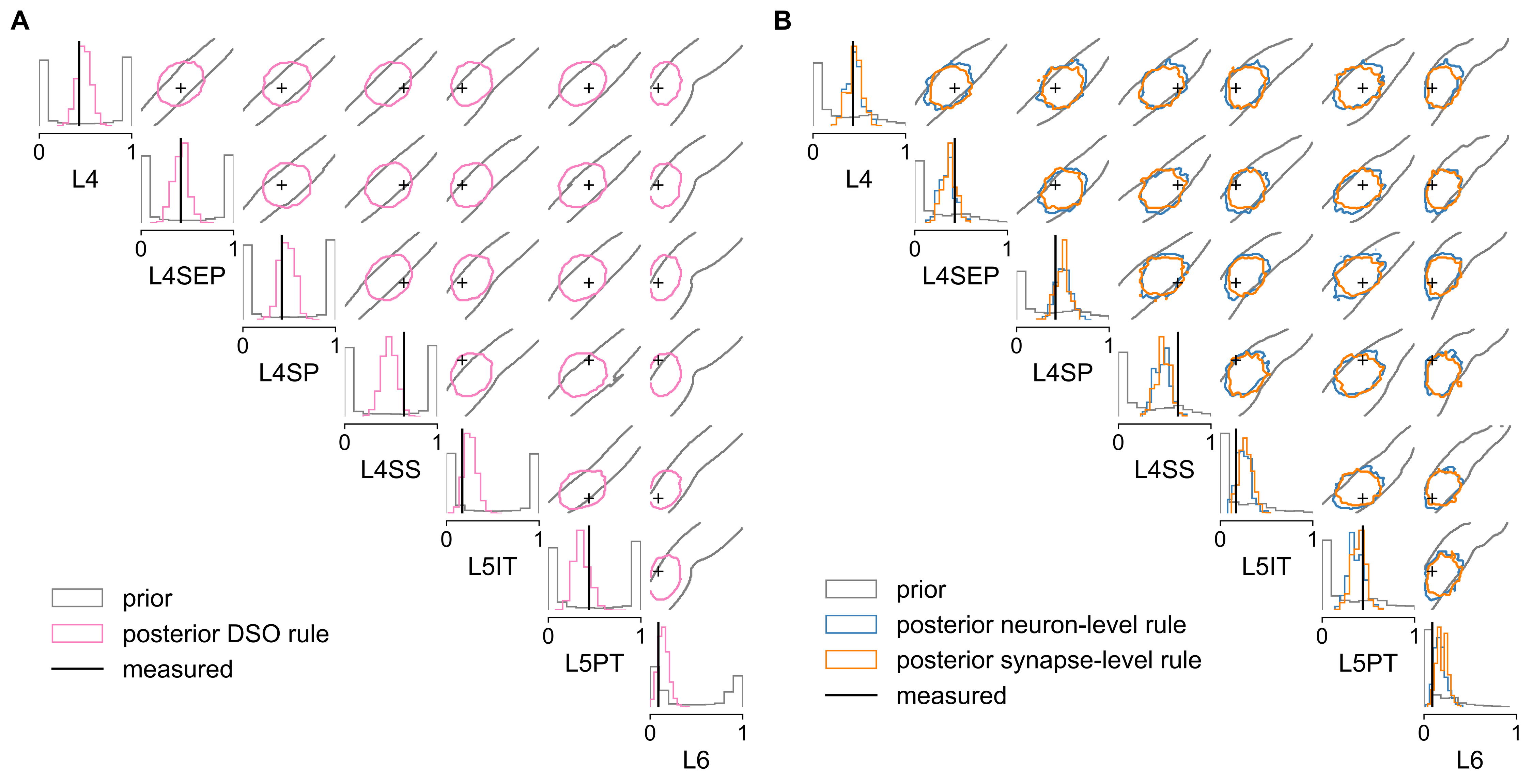

Supplement: S2 Fig — (TIF) [file pcbi.1011406.s002.tif]

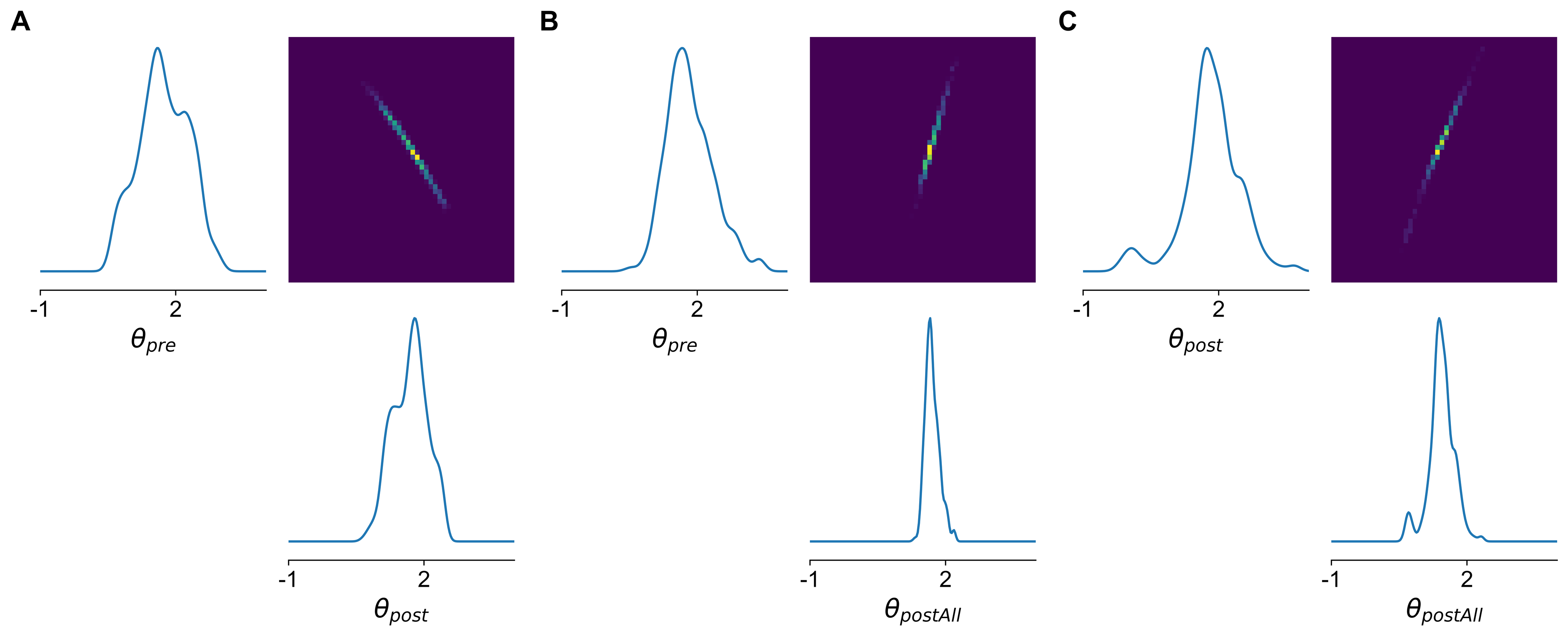

Supplement: S3 Fig — (TIF) [file pcbi.1011406.s003.tif]

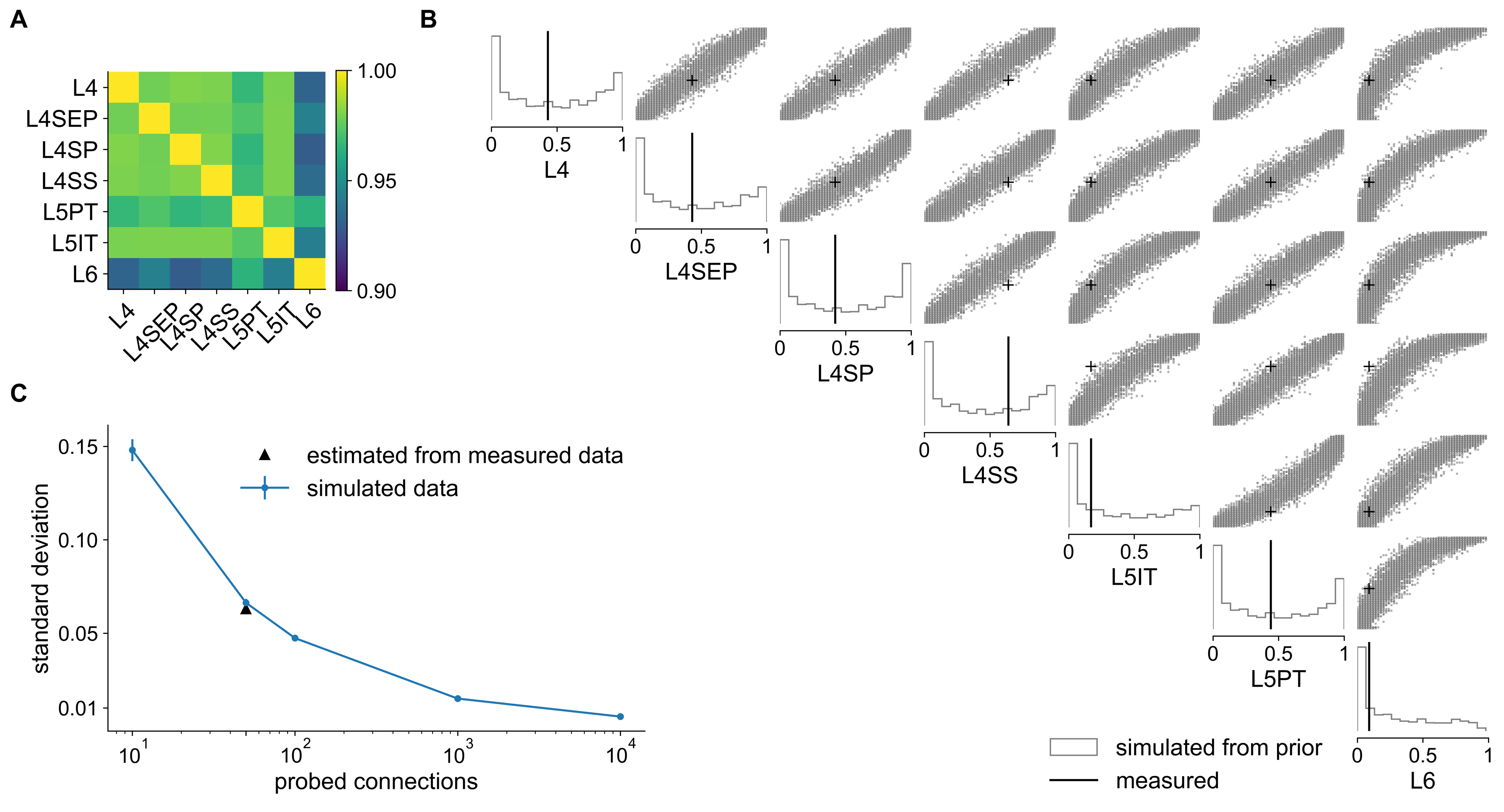

Supplement: S4 Fig — (TIF) [file pcbi.1011406.s004.tif]

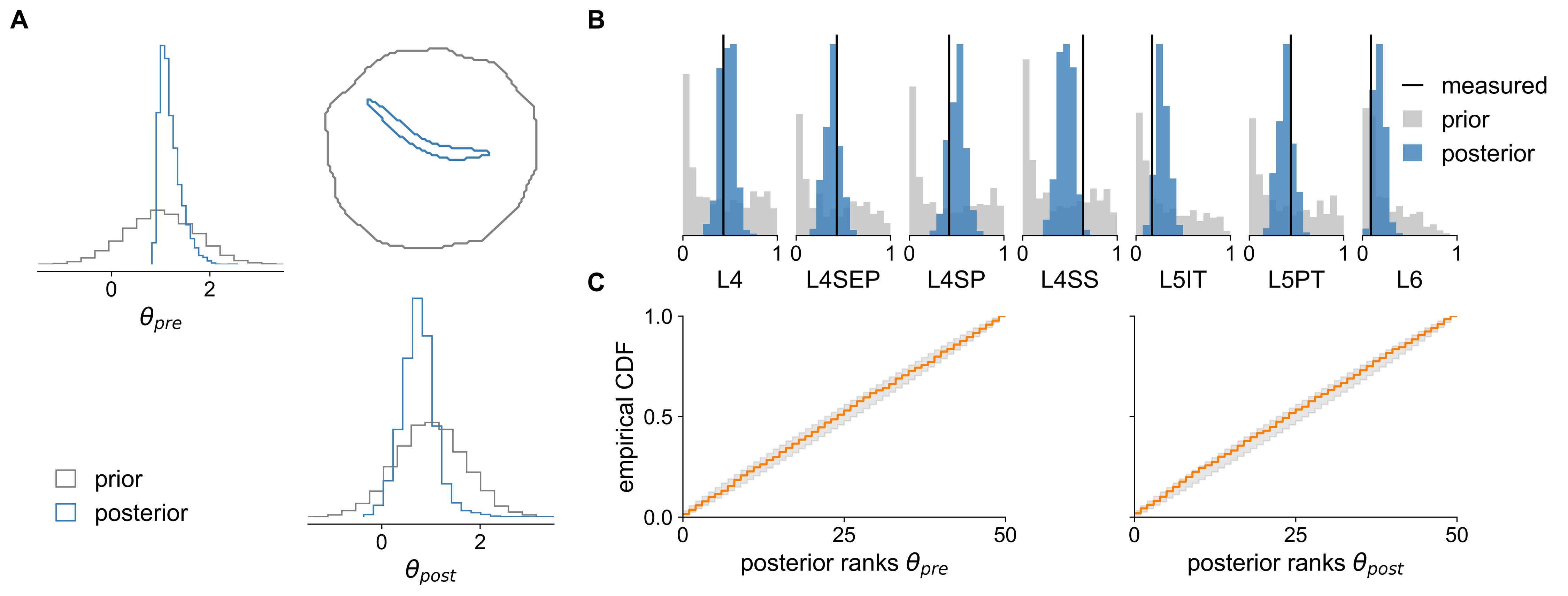

Supplement: S5 Fig — (TIF) [file pcbi.1011406.s005.tif]
