# Supplementary material for: Simulation-based inference for efficient identification of generative models in computational connectomics
Source: PLoS Comput Biol. 2023 Sep 22;19(9):e1011406. doi: 10.1371/journal.pcbi.1011406 (PMC10550169; doi:10.1371/journal.pcbi.1011406)
Supplement: S1 Text — (PDF) [file pcbi.1011406.s006.pdf]

S1 Text

Prior predictive checks

An essential requirement for a generative model is that it can actually generate the measured data given parameter sampled from the prior distribution, i.e., that the model is not misspecified. One way to test for model misspecification is to examine the prior predictive distribution—the distribution of simulated data obtained by running the simulator with parameters sampled from the prior. For the wiring rule simulator, the prior predictive is a seven-dimensional distribution (S4 Fig).

We found that with the chosen setting of the prior, simulator, and summary statistics, it depended on two factors whether the prior predictive distribution included the measured data: the prior covariance σ, and whether the calculation of the summary statistics accurately matches the experimental settings used to obtain measurements. By increasing the prior covariance one can generate a broader prior predictive distribution covering the entire range of connection probabilities. However, by design of the DSO rule, a larger covariance also resulted in a predictive distribution that is heavily skewed towards extreme values. We found that a value of $\sigma = 0.05$ provided a good trade-off between obtaining a broad prior predictive distribution with moderate skewness.

The other essential factor was the calculation of the summary statistics. We obtained simulated pendants to the measured connection probabilities by averaging over connected pairs in the corresponding indices of the simulated barrel cortex connectome (see Algorithm 2). However, the model provided access to thousands of neuron pairs for each measured cell type. If we took the average over this large number of neuron pairs the resulting across simulations was very small. Thus, it was essential to match the sample size used in the experiments when selecting neuron pairs to match the corresponding expected standard deviation (S4C Fig). Therefore, when calculating the connection probabilities from the simulated connectome, we selected 50 neuron pairs at random, which corresponds to the number of probes used in the experiments (see main text for details).

Given these prior and simulator settings, the prior predictive distribution covered the entire range of connection probability values and contained the measured data. The two-dimensional marginals show strong positive correlations between all seven connection probabilities and additional blocks of stronger correlations within layer 4 (L4, L4SEP, L4SP, L4SS) and layer 5 (L5PT, L5IT), visible in the correlations matrix (S4A Fig). These correlations were plausible because all populations share the same source populations in the thalamus, and the blocks of stronger correlations correspond to connection probabilities within the same target layer in the cortex. Furthermore, depending on the number of neuron pairs used to calculate connection probabilities (see Mapping from simulated connectomes to measured connectivity data), the simulator accurately matches the empirical variance expected from the measured data (S4C Fig). These results indicated that a subsampling of 50 pairs was adequate to model the experiments.
