# Supplementary material for: Simulation-based inference for efficient identification of generative models in computational connectomics
Source: PLoS Comput Biol. 2023 Sep 22;19(9):e1011406. doi: 10.1371/journal.pcbi.1011406 (PMC10550169; doi:10.1371/journal.pcbi.1011406)
Supplement: S2 Text — (PDF) [file pcbi.1011406.s007.pdf]

S2 Text

Alternative parametrizations of the DSO rule

The strong conditional correlations between the three parameters of the dense structural overlap rule (DSO, see Posterior analysis reveals biologically plausible parameter interactions) indicated that an alternative parametrization of the DSO rule, e.g., using only two parameters, could also be able to explain the measured data. We tested this hypothesis by changing the DSO rule as follows. The initial formulation of the rule is given by

$$\mathrm{DSO}_{i,j,k}(\boldsymbol{\theta}) = \frac{pre_i^{\theta_{pre}} \cdot post_j^{\theta_{post}}}{postAll_k^{\theta_{postAll}}}. \tag{10}$$

The *postAll* structural feature in the denominator contains the post-synaptic target densities of *all* post-synaptic structures in a given subvolume k of the structural model, including the $post_j$ features. It acts as a normalizing factor for the rule, e.g., when the weight θ_{post} increases, a corresponding increase in $\theta_{postAll}$ can maintain a similar overall DSO value. We changed the DSO rule by removing the parameter $\theta_{postAll}$, but including the *scaled* features $post_j^{\theta_{post}}$ into *postAll*, instead of only taking *post*. By taking the scaled version, we maintained the normalizing property of the denominator of the DSO rule: If θ_{post} was high, in the initial three-parameter formulation of the DSO rule, this could be compensated by a corresponding increase in $\theta_{postAll}$. While this was not possible anymore in the two-param version, the compensation occurred implicitly by including the scaled $post_j$ features:

$$\mathrm{DSO}_{i,j,k}(\boldsymbol{\theta}) = \frac{pre_i^{\theta_{pre}} \cdot post_j^{\theta_{post}}}{(post_j^{\theta_{post}} + postAll_{k,\neg j})}. \tag{11}$$

We performed SBI using the same settings as before to obtain the posterior distribution over the two parameters of the reduced DSO rule (S5A Fig). The resulting posterior predictive distribution showed accuracy similar to the one of the three-parameter DSO rule (compare S5B Fig versus Fig 3B). This result indicates that the two-parameter DSO rule provides an alternative to the three-parameter rule.
