# Supplementary material for: Simulation-based inference for efficient identification of generative models in computational connectomics
Source: PLoS Comput Biol. 2023 Sep 22;19(9):e1011406. doi: 10.1371/journal.pcbi.1011406 (PMC10550169; doi:10.1371/journal.pcbi.1011406)
Supplement: Attachment — Submitted filename: SBI-for-Connectomics Response Letter.pdf [file pcbi.1011406.s008.pdf]

EBERHARD KARLS
UNIVERSITÄT
TÜBINGEN

Faculty of Science

Machine Learning in Science, Maria-von-Linden-Straße 6, 72076 Tübingen

Faculty of Science
Department of Computer Science
Machine Learning in Science

Secretary: Franziska Weiler
Maria-von-Linden-Straße 6
72076 Tübingen
Germany
Telefon +49 7071 29 70850
Franziska.Weiler@uni-tuebingen.de

Tübingen, June 26th, 2023

Dear all,

We would like to thank you for your positive feedback and insightful suggestions on our manuscript. We have made substantial revisions to the manuscript to address your concerns. Specifically, we edited the introduction to improve clarity on the difference between our method and existing approaches. We conducted additional experiments with an updated prior distribution and adapted the results section, Figure 2 and Figure 3 to enhance the interpretability of our findings. Furthermore, we extended the discussion to highlight the potential applicability of our method in related modeling domains.

Through these revisions, we believe that our manuscript now offers a more comprehensive and insightful account of our research, and we are confident that these enhancements will significantly strengthen the overall quality of the paper.

We provide a point-by-point response to the individual comments below. *Reviewer comments are copied in italics*, our responses in red, and corresponding changes in the revised manuscript in blue.

With best regards,

Jan Boelts and Jakob Macke, on behalf of all authors.

Reviewer 1

Boelts et al present the application of the simulation-based inference (SBI) framework to generative modeling of connectomes. The authors first introduce the SBI framework as applied to generative modeling, before applying it to formulate wiring rules of synaptic connectivity in rat barrel cortex. In multiple experiments, the authors evaluate SBI accuracy, study interactions between recovered model parameters and demonstrate the use of SBI to reject implausible wiring rules, using a comparison of SBI-inferred parameters to those derived from Peter's rule.

The study is timely, thorough, and nicely illustrated. My main issue is that the methods and results are occasionally difficult to follow, and that additional interpretation of some of the results could be provided. Please find below suggestions and questions aimed at improving clarity.

Dear Reviewer 1,

We sincerely thank you for dedicating your time to reviewing our manuscript and providing us with your thoughtful and constructive comments. We have carefully considered each of your points and have made the necessary revisions to the manuscript. Below, you will find our in-line responses to your comments and questions, accompanied by screenshots of the corresponding manuscript changes.

Comments

1. Figure 1 and parts of the introduction aim to summarise the advantages of SBI over conventional approaches to generative modeling. However, it isn't entirely clear whether the comparison is fair, for example in the comparison of a "single hypothesis about the connectome" in conventional modeling, with "multiple a-priori hypotheses" in SBI (Fig. 1). Conventional generative modeling does enable comparison of multiple competing hypotheses, through implementation of multiple candidate models. Even within a single model, multiple parameter combinations can be systematically evaluated. Similar language is used in the text, when suggesting that "conventional approaches often optimize for one best-fitting parameter". Arguably, the conventional process can be seen as more "manual" and potentially less efficient than the SBI approach. However, the terminology used in the figure and main text seems to oversimplify the comparison.

Thank you for pointing this out. We acknowledge that our previous description may have oversimplified the comparison between SBI and conventional approaches in generative modeling. In the revised version, we changed the wording accordingly. While it is true that conventional parameter fitting algorithms can be used to test multiple hypotheses, we emphasize that Bayesian inference offers additional benefits. Specifically, Bayesian inference reduces the manual effort involved in model fitting, improves efficiency by automatically returning multiple model configurations, and naturally quantifies uncertainty and parameter interactions.
We have updated the third paragraph of the introduction as follows:

Building generative models that accurately reproduce connectivity measurements can be challenging: Suppose a hypothesized wiring rule does not reproduce the data. In that case, a common approach would be to introduce free parameters to the rule and employ a parameter fitting algorithm to find the best-fitting wiring rule parameters (Fig. 1a). However, identifying one specific wiring rule configuration for which simulated and empirical data match might not be sufficient: Given that the available empirical connectivity data is sparse compared to the structural and functional complexity of the connectome, it is likely that there are many data-compatible wiring rules. While some parameter fitting algorithms can be adapted to identify and compare multiple wiring rule configurations as well (see, e.g., ***Liu et al., 2023***), they require a manual assessment of different solutions and generally involve many repetitions of the simulate-and-compare-to-measurements loop, which can be laborious and inefficient..

To address these challenges, we propose a new approach that employs Bayesian inference to enhance identification of generative models in computational connectomics (Fig. 1b) ~~to replace the manual comparison of individual wiring rules with the automated inference of multiple wiring rules.~~ We achieve this by taking two conceptual steps: First, we equip the generative model with parameters $\boldsymbol{\theta}$ and interpret different parameter combinations as variants of the underlying hypothesis, e.g., variants of the wiring rule. Second, we define a probability distribution over the model parameters such that each parameter configuration corresponds to a different candidate wiring rule, i.e., a *prior distribution* $p(\boldsymbol{\theta})$ (Fig. 1b, left), and use Bayesian inference to infer all data-compatible parameters. Given measured connectivity data x_o and a parametrized generative model, we infer the conditional probability distribution over the model parameters given the measured data, i.e., the *posterior distribution* $p(\boldsymbol{\theta}|x_o)$. The posterior distribution automatically characterizes *all* parameter configurations (wiring rule variants) likely to explain the measured data ~~in contrast to conventional approaches that often optimize for one best-fitting parameter~~. For example, by sampling different parameters from the inferred posterior we would obtain different wiring rule configurations all of which are likely to generate data similar to the measured data (Fig. 1b, bottom). Additionally, the posterior distribution naturally quantifies uncertainties and correlations between the parameters, which can help to reveal parameter interactions and potential compensation mechanisms in the model.

2. The methodological details are dense and at times hard to follow, particularly for readers not familiar with Bayesian inference and generative modeling. In particular, the paragraph on validating SBI performance, titled "SBI performs accurately on simulated data" was difficult to follow without reference to both Materials and Methods, and Supplementary Information. I would recommend providing additional details here.

Thank you for bringing this to our attention. We revised the subsection "SBI performs accurately on simulated data" as follows:

SBI performs accurately on simulated data

Before applying SBI to infer the parameters of the DSO rule given measured data, we validated its accuracy on simulated data. As a first step, we considered a reduced version of the DSO rule simulator for which it was possible to obtain a ground-truth reference posterior distribution (see section Methods & Materials for details). Using this reference solution, we checked whether SBI infers the posterior accurately and how many training simulations it requires. We compared three SBI algorithms: Sequential Neural Posterior Estimation (SNPE, ***Papamakarios and Murray, 2016***; ***Lueckmann et al., 2017***; ***Greenberg et al., 2019***), which performs SBI sequentially over multiple rounds focusing inference on one particular observation; its non-sequential variant NPE; and a classical rejection-sampling-based approach called Sequential Monte Carlo (SMC, ***Sisson et al., 2007***; ***Beaumont et al., 2009***). We found that all three methods can accurately infer the reference posterior distribution (Suppl. Fig. S1a,b) but that they differ in terms of simulation efficiency, i.e., how many model simulations are required for accurate inference: SNPE was slightly more efficient than NPE, and both were substantially more efficient than SMC (Suppl. Fig. S1b).

Additionally, we extended the paragraph *preceding* that section to describe our SBI more clearly (see response to point 3 below).

3. When introducing application of the model to rat barrel cortex, the authors describe that "simulating data with random parameters covers the entire range of probabilities […] including the measured data". Perhaps I am missing something, but this seems trivial. Can the authors clarify the aim of this step? Can the authors also comment on the shape of the resulting prior distribution, heavily skewed towards the extreme values (0 and 1)?

Our aim was to choose the prior such that the simulated connection probabilities with parameters sampled from the prior contain the measured connection probabilities, i.e., to perform a prior predictive check. To that end, we generated a large number of simulated connection probabilities and visualized the resulting seven-dimensional prior predictive distribution as a pairplot (see Supplementary Figure S4). On the one-dimensional marginals that we show in Figure 2d as well, it may indeed seem trivial to cover the entire range from 0 to 1. However, on the two-dimensional marginals (Figure S4), we observed that for small prior variances, the measured data is not contained in the prior predictive distribution, i.e., the prior was misspecified, which is a common problem in SBI. During our initial experiments, we therefore increased the prior variance such that the resulting prior predictive distribution contains the measured data.
That said, we agree that our choice of prior covariance for the results presented in the manuscript is indeed relatively broad, resulting in a prior predictive distribution skewed towards extreme values. For the revision, we therefore re-ran our analysis with smaller prior covariances of 0.05 (was 0.5 before), for which the prior predictive distribution marginals were less skewed but still not misspecified.
We updated the main text, Figure 2d and the appendix accordingly:

Our SBI approach can be summarized as follows: Given a generative model that can simulate barrel cortex connectomes from many different scaled variants of the DSO rule, we use SBI to infer those variants that agree with connection probabilities measured in the barrel cortex. To perform SBI, we define a prior distribution $p(\boldsymbol{\theta})$ over the DSO rule parameters and generate training data for SBI by sampling random parameter values from the prior and simulating corresponding connection probabilities (Fig. 2d, see Methods & Materials for details). We selected the prior such that sampling random parameter values from the prior resulted in connection probabilities covering a broad range of values, including the measurements (Fig. 2d, right, see Experimental settings and Prior predictive checks for details). The prior also contained parameter combinations that led to extreme values, e.g., connection probabilities close to zero. SBI leverages this diverse set of parameter-data pairs to learn an approximation to the posterior distribution over DSO parameters $p(\boldsymbol{\theta}|x_o)$ and thereby identifies those parameters that reproduce the measured data.

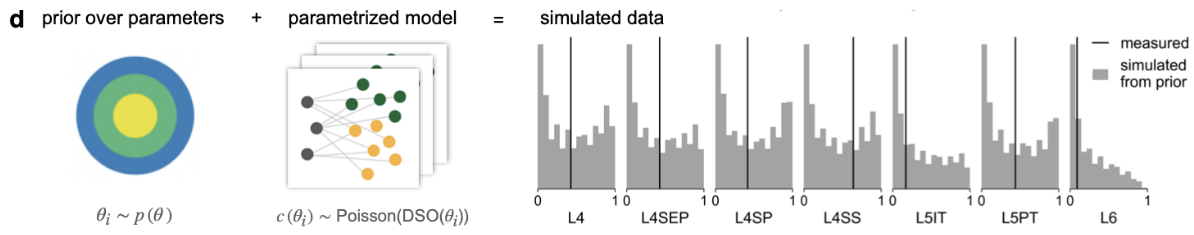

Note that the resulting prior predictive distributions were still skewed towards zero. However, we want to point out that this is due to the design of the DSO rule: For specific parameter combinations, the DSO rule leads to very low connection probabilities (the weights for the pre- and postsynaptic features are small compared to the $\theta^{postAll}$ weight in the denominator leading to a very small DSO value) or very large connection probabilities ($\theta^{postAll}$ is small so that the DSO becomes large). We found that the inferred posterior distributions and predictive distributions were qualitatively similar (see response to point 4 below).

4. The authors apply SBI to simulate connectomes of the rat barrel cortex, based on the “dense structural overlap” (DSO) rule, and identify parameter combinations which are compatible with the observed data. However, an interpretation of these findings seems to be missing. Can the recovered values be interpreted relative to the literature? Does the parametrized DSO rule provide additional insight over the previous form, assuming that pre- and postsynaptic features have the same relative importance? On a related note, it seems notable that the maxima of recovered parameter values in the parametrized DSO rule are highly similar to each other, also suggesting a relatively similar importance of DSO model features.

Thank you for pointing this out. We edited the corresponding sections to improve interpretability of our results. Specifically, we updated Figure 3 and the main text with the results obtained with the updated prior distribution (see response to point 3). We found that the updated prior does not change the inference results qualitatively—we observed a similar correlation structure in the two-dimensional posterior marginals.
We also made several changes regarding the comparison to the initial “a-priori” version of the DSO rule. Specifically, we now provide more details on why the weighting of the DSO rule features could in principle be unequal:

Udvary et al. (*2022*) showed that the DSO wiring rule can reproduce measured network characteristics at different scales. However, in its current form, the DSO rule assumes that the pre- and postsynaptic features have the same relative weight in determining the probability of a synapse and that these weights are the same across all barrel cortex cell types. Is this specific combination of pre- and postsynaptic features in the DSO rule the only valid choice? Specifically, it has been found that single boutons from VPM axons can establish multiple synapses in cortical layer four (***Rodriguez-Moreno et al., 2018***); it has also been observed that in some cases, multiple synapses are formed at a single postsynaptic spine (***Santuy et al., 2018***). Thus, the weighting of the DSO features could, in principle, be unequal. A common approach to testing this question would be to

Additionally, we added a visualization of the “a-priori rule”, both in the parameter space (Fig. 3a, orange dot at center of prior) and in the posterior predictive plot (Fig. 3b, orange histogram):

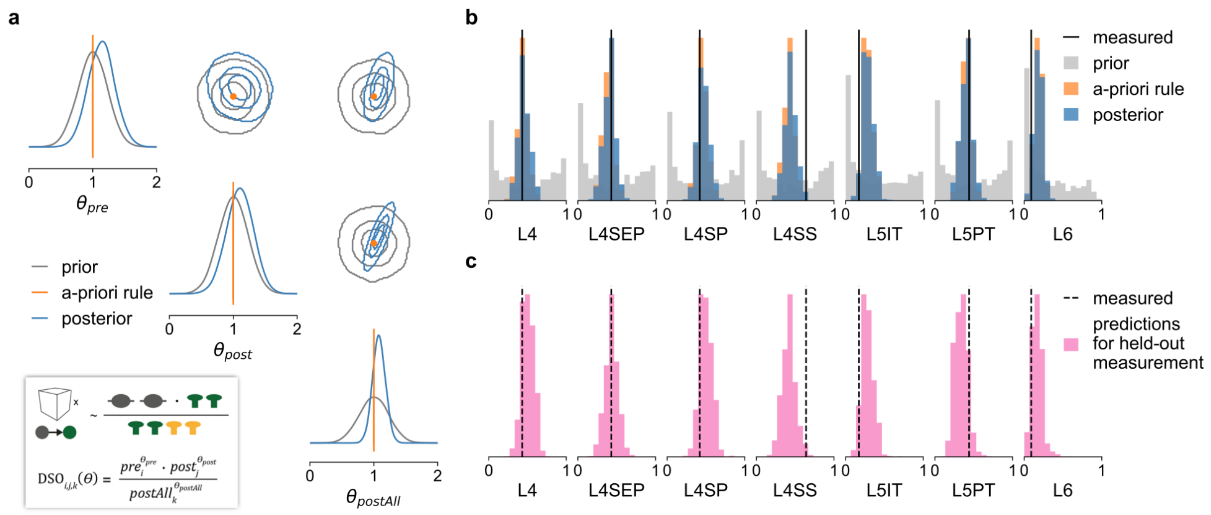

This visualization showed that the initial version and the SBI-inferred version of the DSO rule can explain the measured data similarly well (Fig. 3b), and that the SBI-inferred DSO rule also suggests a relatively similar importance of the DSO features.
That being said, we want to emphasize that the central goal of our work was to show that SBI *can* be applied in this scenario and how the resulting posterior distributions can be analyzed (rather than investigating whether the parametrized DSO rule provides additional insights). The crucial benefit of the SBI approach is that it shows that there are many variations of the parametrized DSO rule that can reproduce the measurements equally well, and that it reveals the corresponding correlations structure of the parameter space.
We updated the main text as follows:

The posterior identifies many data-compatible wiring rule configurations.

We found that the inferred posterior distribution was relatively broad, indicating that there are many parameter combinations with a high probability of explaining the measured data. The most likely parameter combination, i.e., the maximum of the one-dimensional posterior marginal distributions (Fig. 3a, blue in diagonal subplots) was $\theta_{MAP} = [1.14, 1.1, 1.08]$, corresponding to a relatively

similar weighting of pre- and postsynaptic DSO rule features. This was in line with the assumption of the initial "a-priori" version of the DSO rule ($\boldsymbol{\theta} = [1, 1, 1]$, orange lines in Fig. 3a). Importantly, however, the posterior marginal standard deviations of $\hat{\sigma}_{pre} = 0.2$, $\hat{\sigma}_{post} = 0.2$ and $\hat{\sigma}_{postAll} = 0.1$ showed that this is not the only solution but that there are several other parameter combinations with high posterior probability. For example, sampling parameter values from the posterior for θ_{post} would return values lying mostly in an interval as broad as [0.7, 1.5] ~~[0.3, 1.69]~~ (95% posterior credible interval). Despite this relatively broad range of plausible parameters values, we still found that the posterior predictive distribution, i.e., connection probabilities simulated with parameters sampled from the posterior, clustered around the measured data and closely matched the data simulated from the initial version of the DSO rule (Fig. 3b). Quantitatively, we observed that all except the L4SS and L5IT measurements were located within one standard deviation of the posterior predictive distribution, which matched the standard deviation expected from the sample size used in the experiments (***Bruno and Sakmann, 2006***; ***Constantinople and Bruno, 2013***, see Methods & Materials for details). How can so many parameter configurations from such broad ranges all result in similar data?

Additionally, we provide a more detailed interpretation of the results at the end of the subsection:

Collectively, the SBI approach enabled us to efficiently identify several different DSO rule configurations that can all explain the measurements, among them the initially proposed version of the DSO rule. The analysis of the inferred posterior showed that the number of presynaptic boutons and the number of postsynaptic contact sites (and, by extension, axonal and dendritic path length) are sensitive and strongly interdependent structural features for predicting synaptic connectivity. Further, we found that the inferred parameters attribute similar weights to the DSO rule features and could predict the measurements well (except for the L4SS and L5IT cell types), indicating that multi-synaptic contacts at pre- or postsynaptic sites (***Rodriguez-Moreno et al., 2018***; ***Santuy et al., 2018***) do not significantly impact the predictions of the DSO rule. The mismatch between the predicted and measured connection probabilities that we found for the L4SS and L5IT cell types (Fig. 3b) suggests the DSO-rule assumption of similar scaling of DSO features does not hold here. Applying the SBI approach to an extension of the DSO rule with additional features or separate scaling factors for these cell types would likely reproduce these measurements more accurately.

5. Further, the authors use held-out data to predict unobserved connection probabilities. The description and interpretation of some aspects of this experiment could be more extensive. For example, authors note that "predictions […] clustered around actual measurement values for most of the seven connection probabilities". Statistics would be beneficial here, to quantitatively compare alignment of measures and predicted parameters. Moreover, the predictions for some neurons seem poorly aligned with measurements; a more detailed discussion of this, and of the differences in agreement between measured and predicted parameters across neuron types, would be welcome.

Thank you for pointing this out. We agree that a quantification and a more detailed discussion of the posterior predictions was missing in the initial version of the manuscript. We quantified the match between posterior predictive distributions and measurements with respect to the measurement noise we would expect from the experimental setup. Given the sample size of around 40-50 paired recordings used by Bruno et al. and Constantinople et al., one can expect a standard deviation in the estimated connection probabilities of around

0.08. We found that for all except the L4SS and L5IT cell types, the mismatch between the measurement and the posterior predictive mean was smaller than 0.08. We added these quantifications to the revised version of the manuscript and provided a more detailed discussion on the mismatch for L4SS and L5IT (see screenshots of manuscript changes under point 4).

6. Finally, the authors briefly mention the use of a classifier trained to distinguish between predictions inferred from all measurements, and for held-out measurements. More details should be provided here, on the aims of this, the classifier used, and interpretation of the findings (i.e. held-out predictions seemingly failing for some neuron types).

The aim of this step was to show that the posterior predictive distribution can be used to make predictions for future experiments, e.g., predict the effect of the DSO rule for cell-type populations in the barrel cortex that have not been measured yet. To emulate this scenario, we trained the SBI posterior estimator on a subset of the connection probabilities, i.e., we held out one of the seven connection probabilities. After training, we obtained posterior samples, simulated all seven connection probabilities and thereby predicted the outcome of the held-out measurement.
To quantify the accuracy of this prediction, we compared posterior predictive distributions obtained from a SBI posterior-estimator trained with incomplete data with the one obtained with access to all seven measurements using a classifier-two-sample-test (C2ST). Concretely, we trained an artificial neural network-based classifier to classify predictive samples from the posterior trained on all seven measurements (class 1) and predictive samples from the posterior trained on only six measurements (class 2). The classification test-accuracy between class 1 and 2 then indicates how similar the two sets of samples (posterior predictive distributions) are. For example, if it is close to 0.5, this indicates that they are indistinguishable (given the classifier has enough capacity and has converged). We found that for all but the L4SS and the L5PT cell types the C2ST scores were below 0.55. This indicated that for each of those well-predicted cell types, the weighting of the DSO rule features inferred for the other cell types also applies to this cell type. Additionally, it indicates that the weighting of L4SS and L5PT cannot be predicted from the weightings inferred from the other cell types, in line with the overall worse predictive performance for these cell types.
In the updated version of the manuscript, we extended our explanation of the C2ST classifier analysis including the above interpretations:

The posterior predictive distributions for held-out measurements clustered around the actual measurement values for all of the seven cell types, except for L4SS and L5IT (Fig 3c), and closely resembled the predictive distributions of the posterior inferred given all measurements (Fig 3b). To quantify this, we performed a classifier-based two-sample test (C2ST, ***Lopez-Paz and Oquab, 2018***; ***Lueckmann et al., 2021***): We trained a two-layer artificial neural network to classify the predictions of the posterior inferred using all measurements (class 1) from the predictions of the posterior

inferred using only six measurements (class 2). In this setting, a classification accuracy close to 0.5 indicates performance at chance level, i.e., the two classes are indistinguishable. We found that the C2ST accuracy was below 0.55 for all measurements except for L4SS (0.68) and L5PT (0.59). This indicated that for all but the L4SS and L5PT cell types, the weighting of the DSO features could be accurately predicted from the other cell types, in line with the results presented above. Collectively, these cross-validation results suggested that the structural model paired with the SBI-enabled wiring rule enables us to make experimentally testable predictions. For example, one could predict connection probabilities of cell types different from the seven measured here or other connectivity features of the rat barrel cortex available in the structural model (see below).

7. Similarly to above: during evaluation of the neuron-level and synapse-level rules, the measurement for the L4SS neuron is better aligned with Peter's rule, than with the inferred data. It would be useful to quantify this (mis-)alignment, and certainly to discuss and interpret it.

Indeed, for the L4SS cell type the predictions of Peters' rule are better aligned with the measurements than the inferred DSO rule. We updated the main text to quantify and discuss this mismatch as follows:

When applying Peters' rule to the barrel cortex model at the neuron level, a connection occurs whenever two neurons share at least one subvolume, i.e., the connection threshold would be $\theta_{thres} = 1$. Does this assumption hold for the barrel cortex at the neuron-to-neuron level as well? To answer this question quantitatively, we used SBI to infer the threshold parameter θ_{thres} of the neuron level rule for each edge length. We observed that the inferred threshold parameters shifted to larger values with increasing edge length, e.g., the higher the spatial resolution, the fewer common subvolumes were required to obtain a connection (Fig. 4b). This was in line with our observation that the overall number of shared subvolumes available in the structural model increased with increasing edge length (Fig. 4a). However, irrespective of the spatial resolution, all inferred threshold parameters were substantially larger than the $\theta_{thres} = 1$ of Peters' rule, reaching from $\theta_{thres} \approx 3$ (posterior mean) for the $1\,\mu m$-subvolume model (Fig. 4b, orange) to $\theta_{thres} \approx 28$ for the $50\,\mu m$-subvolume model (Fig. 4b, violet). Accordingly, the comparison of predictive performances showed that for all but the L4SS cell type, the difference between measurement and the average prediction was smaller for the inferred rule than for Peters' rule and below one standard deviation expected in the experiments (Fig. 4c). For the L4SS cell type, we found that data simulated with Peters' rule was close to the measurement (0.02 difference).

Nevertheless, we want to emphasize that despite this matching prediction for the L4SS cell type, our analysis showed (confirming previous findings) that Peters' rule systematically overestimates connectivity (see predictions for all other cell types Fig. 4c, and number of predicted synapses in Fig. 5b).

Minor comments

The use of the term "neural networks" in the abstract might be prone to misinterpretation as relating the machine learning context of artificial neural networks, rather than the desired biological context. Perhaps a term such as "neuronal networks" or "connectomes" might be preferred?

The authors share code related to this paper on GitHub, including tutorials, which is welcome. This builds on the prior release of the “sbi” Python package, which is a laudable effort. However, the authors might wish to rephrase the last sentence of the introduction, to clarify that the “sbi” package is not being made available for the first time in this context.

Thank you for the additional points. We decided to use “neuronal networks” in the abstract and changed the last sentence of the introduction to credit the SBI package appropriately:

> *2022*) and inferring underlying connectivity principles using SBI. We are making all software tools required for applying SBI available via an established open-source software package for SBI (`sbi`, *Tejero-Cantero* et al., 2020*), facilitating its use by researchers across the field.

Typos

*“Is this specific combination of pre- and postsynaptic features in the DSO rule *is* the only valid choice?”*

*“…the higher *the* spatial resolution, the fewer common subvolumes were required…”*

Reviewer 2

Thank you for the opportunity to review this work by Boelts and colleagues. The authors propose an interesting use of simulation-based inference (SBI) to efficiently fit parameters of connectome generative models. The paper tackles an important issue: fitting wiring rules to empirical connectivity data typically entails time-consuming sweeps of large parameter spaces. Using a combination of Bayesian inference and machine learning, the authors show—through multiple lines of evidence—that their approach can alleviate these costs by simultaneously characterising the posterior distributions of all wiring parameters.

My opinion of the work is positive. The paper was a pleasure to read—the motivation for the study is set out clearly and didactically, the figures are beautiful and informative, and the text is very well written. That said, I do have a few comments and questions below. My expertise is in network neuroscience and I have only superficial knowledge in Bayesian inference and machine learning, so most of my remarks are related to the application of the present framework to the type of generative models done in network neuroscience. I hope the authors will find these comments useful to further improve their work.

Dear Reviewer 2,

Thank you very much for taking the time to review our manuscript! We are glad that your overall impression is positive. Thank you also for your constructive comments, questions and suggestions. We believe that your input from the network neuroscience perspective substantially improves the applicability of our work.
Below, you find responses to each of your points (in red) and the corresponding changes made to the manuscript as screenshots.

1. One of the key steps in the wiring rule simulator is "3. Calculation of summary statistics from the simulated connectome to match the available measurements". Here, the authors analysed a cellular-level network of the rat barrel cortex, and the fit to empirical data was measured as the ability to recapitulate observed connection probabilities between different cell types. Instead, in the network neuroscience literature (e.g., Betzel 2016, Vertes 2012; all properly cited), the typical goal is to match complex topological properties of empirical connectivity. That is, to generate synthetic networks with, e.g., degree, connection-centrality, or connection-length distributions that match those observed in empirical brain networks. In many cases, researchers seek to match multiple properties at once, and do so by defining multi-objective energy functions. Can the SBI framework be applied in these—arguably—more complex scenarios? The computation of these network measures can be time consuming, and I imagine it could pose certain barriers to the estimation of parameter posterior distributions? To be clear, I think the present focus on cell type connection probabilities is perfectly fine, I am just trying to gauge the applicability of this framework to different lines of connectome research. I would appreciate it if the authors could comment on this point.

Thank you for making this important point. Yes, SBI can be applied to more complex scenarios than presented in the paper, but it depends on the problem at hand. In principle, SBI can be applied to any scenario in which we have empirical data and a simulator that can generate corresponding synthetic data. For example, in a network neuroscience application,

one could define the generation of synthetic networks from a parameterized model and the subsequent computation of network measures as one simulation, and then perform SBI to constrain the model parameters with empirical network measures. Whether this is feasible in practice depends—as you suggested—on the complexity of the inference problem (e.g., number of model parameters) and on how much training data we can generate (simulation time). In our wiring rule example, we had only three model parameters and the simulation time was about 1sec, which one could consider an “easy” SBI problem. Currently available SBI methods have been successfully applied in scenarios with up to 50 model parameters and simulation time on the order of seconds.

In the revised version of the manuscript we added more details on this point in the discussion:

Applicability and limitations

In this work, we applied SBI to the specific problem of inferring wiring rules that can generate observed connectivity data. A key advantage of SBI is that it is generally applicable to any computational model capable of simulating data from a set of parameters, i.e., only data simulated from the model are required to infer the posterior distribution over model parameters. For example, one could make the algorithms for generating synthetic functional connectomes proposed by ***Vértes et al.*** (***2012***) or ***Klimm et al.*** (***2014***) amenable to SBI by introducing parameters of interests and then use SBI to efficiently identify all data-compatible parameter values. Similarly, SBI could be applied to infer unknown parameters of iterative growth-based generative models as used in the works by ***Betzel et al.*** (***2016***); ***Oldham et al.*** (***2022***); ***Liu et al.*** (***2023***). Concretely, one would generate many different synthetic networks from a given growth model using many candidate parameter settings and then use the simulated data in the SBI framework to obtain the posterior distribution given empirical network data.

[...]

SBI’s dependency on simulated data and neural network training also entails several limitations: the inferred posterior distributions are only approximations of the unknown actual posterior distribution. Therefore, applying SBI requires careful evaluation of every problem at hand. In theory, SBI does recover the unknown posterior distribution when given enough training data (***Papamakarios and Murray, 2016***). In practice, however, the accuracy and reliability of SBI strongly depend on the complexity of the inference problem, e.g., on the number of model parameters, the complexity of the data, and the simulation time. Previous studies have successfully applied SBI in scenarios where the simulator has a runtime on the order of seconds and up to thirty parameters (see ***Gonçalves et al., 2020***; ***Deistler et al., 2022b***; ***Ramesh et al., 2022***, for examples), but these numbers strongly depend on the problem and available computational resources.

2. Is there a distinction to be made here between estimating all parameter values for a single wiring rule vs. finding all potential wiring rules? In several places in the manuscript, including the author summary (lines 45–47), it is implied that these two things are the same. From my understanding, SBI allows for an efficient exploration of all parameter values for a pre-specified wiring rule. I do not think the authors provided any evidence that their framework allows for a simultaneous exploration of all possible wiring rules—indeed, that would be very hard, as there are seemingly infinite ways to formulate a wiring probability equation. To come back to the network neuroscience literature, a lot of work has been done comparing different wiring rules, i.e., connectivity growth equations constructed based on different topological properties of the networks. I currently don't see how SBI could handle

the automation of this comparison. Could the authors comment on this?

Thank you for pointing this out. Indeed, an important distinction has to be made: We used SBI to infer the parameters of one specific predefined wiring rule and it would be infeasible to use SBI for exploring all possible different wiring rules simultaneously. We agree that the initial version of the manuscript should be more precise on this point and we updated it accordingly. For example, in the abstract, we changed lines 24-26 to

simulation-based Bayesian inference (SBI) to address this challenge. Rather than optimizing a fixed wiring rule to fit the empirical data, SBI considers many parametrizations of a rule and performs Bayesian inference to identify the parameters that are compatible with the data. It uses simulated data from multiple candidate wiring rule parameters and relies on machine learning methods to estimate a probability distribution (the 'posterior distribution over parameters conditioned on the data') that characterizes all data-compatible ~~rules~~parameters. We

In the author summary we now write

Author summary

The brain is composed of an intricately connected network of cells—what are the principles that contribute to constructing these patterns of connectivity, and how? To answer these questions, amassing connectivity data alone is not enough. We must also be able to efficiently develop and test our ideas about the underlying connectivity principles. For example, we could simulate a hypothetical wiring rule like "neurons near each other are more likely to form connections" in a computational model and generate corresponding synthetic data. If the synthetic, simulated data resembles the real, measured data, then we have some confidence that our hypotheses might be correct. However, the proposed wiring rules usually have unknown parameters that we need to "tune" such that simulated data matches the measurements. The central challenge thus lies in finding all the potential parametrizations of a wiring rule~~, or equivalently, all the parameters of the computational model~~ that can reproduce the measured data, as this process is often idiosyncratic and labor-intensive. To tackle this challenge, we introduce an approach that combines computational modeling in connectomics, deep learning, and Bayesian statistical inference in order to automatically infer a probability distribution over the model parameters likely to explain the data. We demonstrate our approach by inferring the parameters of a wiring rule in a detailed model of the rat barrel cortex and find that the inferred distribution identifies multiple data-compatible model parameters, reveals biologically plausible parameter interactions, and allows us to make experimentally testable predictions.

Finally, we revised the introduction to emphasize that we perform inference over the parameters of one particular wiring rule, not over different wiring rules.

That said, there are ways to use SBI for comparing different models (wiring rules) as well. One naive way would be to apply SBI to each model and then compare the predictive performance of the posteriors of the competing models. For a more quantitative comparison, one could apply Bayesian model comparison, i.e., inferring the posterior distribution over models p(M | x) (not over parameters, the model parameters are marginalized out). There are SBI methods available for performing Bayesian model comparison. Essentially, one would then simulate data from all competing models and train a neural density estimator to approximate the (discrete) posterior over models. After training, one can apply the estimator to empirical data to identify the most likely model and, finally, perform SBI on the

parameter-level to obtain the posterior over model parameters. In the current version of the manuscript, we discussed this only briefly. In the revised manuscript we now elaborate on this point when discussing related work:

Bayesian inference methods have been employed in computational connectomics before, but in different ways than the SBI approach proposed here. ***Jonas and Kording*** (***2015***) built a probabilistic model of cell type-dependent connectivity in the mouse retina and proposed a non-parametric Bayesian algorithm that automatically predicts cell types and microcircuitry from connectomics data. More closely related to our approach, ***Klinger et al.*** (***2021***) performed Bayesian model comparison using a rejection-sampling approach (***Toni et al., 2009***) to compare a set of competing local circuit models in layer 4 of the mouse primary somatosensory cortex based on structural connectomics data. However, in contrast to our approach that infers the posterior distribution *over parameters* of one particular model, ***Klinger et al.*** (***2021***) inferred the posterior probabilities *over several models* (with the parameters of individual models integrated out). Moreover, their approach relied on classical rejection-sampling techniques that are less simulation-efficient compared to the neural-network-based SBI we employed and will likely not scale to higher-dimensional inference problems (see Figure S1b and ***Lueckmann et al., 2021***, for a detailed comparison). Yet, there have been recent advances in neural network-based Bayesian model comparison (***Boelts et al., 2019***; ***Radev et al., 2021***; ***Mancini et al., 2022***) and simultaneous model and parameter inference using SBI (***Schröder and Macke, 2023***). Thus, a promising direction for future research in connectomics would be combining Bayesian model comparison and parameter inference, e.g., to compare competing wiring rule hypotheses on the basis of empirical data: One would simulate data from each wiring rule and use SBI to approximate the discrete posterior distribution over different wiring rules to select the wiring rule with the highest posterior probability and simultaneously use SBI to constrain the parameters of the selected rule (as proposed here).

3. Another important point is that the current generative model does not seem to be an iterative growth model. In many network-based generative models, connections are added one at a time. This is important because as the network topology evolves, the addition of each new connection influences the probability of wiring between nodes. In many lines of research this is an important feature of connectome generative models, and it provides an opportunity to study questions relating to biological development over time (see DOI:10.1126/sciadv.abm6127, for example).

We agree, the wiring rules we used are not iterative and that iterative growth model can be seen as more realistic. However, we want to point out that our manuscript focuses on the SBI approach and its successful application to generative models in connectomics in general, and not on proposing a neurobiological model that is as realistic as possible. That said, SBI could be applied to such an iterative growth model too: One would define the parametrized growth model as the simulator, use it to generate synthetic training data, and then perform SBI to constrain the growth model parameters with empirical data.
We thank the reviewer for their reference to doi:10.1126/sciadv.abm6127 — we added it to our list of potential applications of SBI:

model are required to infer the posterior distribution over model parameters. For example, one could make the algorithms for generating synthetic functional connectomes proposed by ***Vértes et al.*** (***2012***) or ***Klimm et al.*** (***2014***) amenable to SBI by introducing parameters of interests and then use SBI to efficiently identify all data-compatible parameter values. Similarly, SBI could be applied to infer unknown parameters of iterative growth-based generative models as used in ***Betzel et al.*** (***2016***); ***Oldham et al.*** (***2022***); ***Liu et al.*** (***2023***). Concretely, one would generate many different synthetic networks from a given growth-model using many candidate parameters settings and then use the simulated data in the SBI framework to obtain the posterior distribution given empirical network data.

4. Might be worth mentioning that virtually all previous work on human connectome generative models also provide evidence against Peters' rule—that is, adding topological information into the wiring rule almost always improves on a naive model based solely on inter-regional distances.

Thank you for pointing this out—we extended the corresponding sentence accordingly:

Using SBI to rule out invalid wiring hypotheses

Above, we demonstrated that SBI provides a quantitative way to identify valid wiring rule configurations from a large set of hypothesized wiring rules. SBI can also be used to rule out invalid hypotheses, e.g., to show that an existing hypothesis does not agree with empirical data. One such debated hypothesis in connectomics is the so-called *Peters' rule* (***Peters and Feldman, 1976***; ***Braitenberg and Schüz, 1991***). According to this hypothesis, neurons form connections whenever their axons and dendrites are in close proximity, i.e., Peters' rule can be formulated as "axo-dendritic proximity predicts connectivity" (***Udvary et al., 2022***). However, several empirical and theoretical studies on rat, mouse and human connectomes found substantial evidence against Peters' rule (e.g., ***Mishchenko et al., 2010***; ***Kasthuri et al., 2015***; ***Rees et al., 2017***; ***Udvary et al., 2022***).

5. A recent reference that should be discussed:
https://www.sciencedirect.com/science/article/pii/S1053811923001088

Thank you for pointing us to this relevant work. We now include it in our discussion of related work:

Related work

The problem of identifying parameters of computational models that reproduce experimentally observed data has been addressed in computational connectomics before. For example, ***Vértes et al.*** (***2012***) built a model of functional connections between brain regions and used optimization methods to find single best-fitting parameters capturing the functional MRI data measured in humans. ***Klimm et al.*** (***2014***) and ***Betzel et al.*** (***2016***) used Monte Carlo sampling methods for optimizing the parameters of synthetic networks of structural connectivity to match the topological properties of human connectomes recorded with MRI. Recently, ***Liu et al.*** (***2023***) proposed a parameter estimation method for connectome generative models based on averaging over ensembles of best-fitting parameters. In contrast to our approach, these studies do not perform Bayesian inference but rely on optimization techniques that identify best-fitting solutions.
